# Adult Mouse Retina Explants: From *ex vivo* to *in vivo* Model of Central Nervous System Injuries

**DOI:** 10.3389/fnmol.2020.599948

**Published:** 2020-11-25

**Authors:** Julia Schaeffer, Céline Delpech, Floriane Albert, Stephane Belin, Homaira Nawabi

**Affiliations:** Université Grenoble Alpes, Inserm, U 1216, Grenoble Institut Neurosciences, Grenoble, France

**Keywords:** central nervous system, axon regeneration, explants, *ex vivo*, optic nerve, retinal ganglion cells, growth cone, axonal transport

## Abstract

In mammals, adult neurons fail to regenerate following any insult to adult central nervous system (CNS), which leads to a permanent and irreversible loss of motor and cognitive functions. For a long time, much effort has been deployed to uncover mechanisms of axon regeneration in the CNS. Even if some cases of functional recovery have been reported, there is still a discrepancy regarding the functionality of a neuronal circuit upon lesion. Today, there is a need not only to identify new molecules implicated in adult CNS axon regeneration, but also to decipher the fine molecular mechanisms associated with regeneration failure. Here, we propose to use cultures of adult retina explants to study all molecular and cellular mechanisms that occur during CNS regeneration. We show that adult retinal explant cultures have the advantages to (i) recapitulate all the features observed *in vivo*, including axon regeneration induced by intrinsic factors, and (ii) be an *ex vivo* set-up with high accessibility and many downstream applications. Thanks to several examples, we demonstrate that adult explants can be used to address many questions, such as axon guidance, growth cone formation and cytoskeleton dynamics. Using laser guided ablation of a single axon, axonal injury can be performed at a single axon level, which allows to record early and late molecular events that occur after the lesion. Our model is the ideal tool to study all molecular and cellular events that occur during CNS regeneration at a single-axon level, which is currently not doable *in vivo*. It is extremely valuable to address unanswered questions of neuroprotection and neuroregeneration in the context of CNS lesion and neurodegenerative diseases.

## Background

For decades, many efforts have been deployed to unlock the cellular programs to achieve axon regeneration in the central nervous system (CNS). Indeed, unlike neurons from the peripheral nervous system (PNS), CNS neurons are not able to grow axons after injury. This regeneration failure is due to the generation of a growth-inhibitory environment at the lesion site (Silver and Miller, 2004; Yiu and He, 2006) and to the loss of intrinsic regrowth ability in mature axons (He and Jin, 2016). Additionally, pro-regenerative molecular pathways are switched off after injury, inhibiting further axon regrowth potential (Park et al., 2008; Belin et al., 2015). Therefore, the activation of developmentally regulated pathways such as mTOR (mechanistic target of rapamycin) (Park et al., 2008), or transcription factors such as KLF (krüppel like factors) (Moore et al., 2009) promotes axon regeneration in different models of CNS injury, such as optic nerve or spinal cord injuries (Park et al., 2008; Moore et al., 2009; Liu et al., 2010; Blackmore et al., 2012). Moreover, as the lesion itself modulates several signaling pathways, their synergistic manipulation leads to long distance regeneration (de Lima et al., 2012; Belin et al., 2015).

Even though many exciting candidates regulating axon regeneration have been uncovered, their modulation in a therapeutic approach remains difficult. Indeed, most of these molecules trigger numerous functions in cells and current knowledge is insufficient to understand which one is essential for axon regeneration. In addition, these regenerative molecules are also known to be oncogenic factors (Liu and Sabatini, 2020). Thus, it is urgent to unravel the precise molecular and cellular events allowing axon regeneration in mature CNS in order to (i) characterize new cellular targets implicated in axon regeneration mechanisms, and (ii) develop innovative therapeutic strategies for CNS repair after a traumatic lesion or in neurodegenerative diseases. In this regard, embryonic cortical or hippocampal neuronal cultures are commonly used as experimental models (Blackmore et al., 2012; Gomis-Rüth et al., 2014). However, while it is easy to obtain a large quantity of isolated neurons in culture, these *in vitro* models cannot answer precisely to the question of axon regeneration in the mature CNS. Indeed, unlike mature neurons, embryonic neurons or young neurons (until P5-P6 in mice) have a high regrowth potential (Martin et al., 2000; Goldberg et al., 2002). For this reason, it is crucial to develop appropriate *in vitro* assays that recapitulate *in vivo* features of mature neurons.

The optic nerve is a relevant model to address the molecular and cellular mechanisms of CNS axon regeneration in adult. Most of the molecular pathways that have been uncovered using optic nerve lesion as a model of CNS injury also show promising results for regeneration in the corticospinal tract (Liu et al., 2010; Jin et al., 2015). Within the retina, only the retinal ganglion cells (RGC) population project their axons to form the optic nerve. These neurons connect directly to functional nuclei of the visual system in the brain. Unlike spinal cord lesions that affect multiple neuronal populations, the optic nerve injury affects only the population of RGC. This unique feature allows to focus specifically on the specific behavior of this population of neurons upon axon injury (Belin et al., 2015).

**FIGURE 1:**
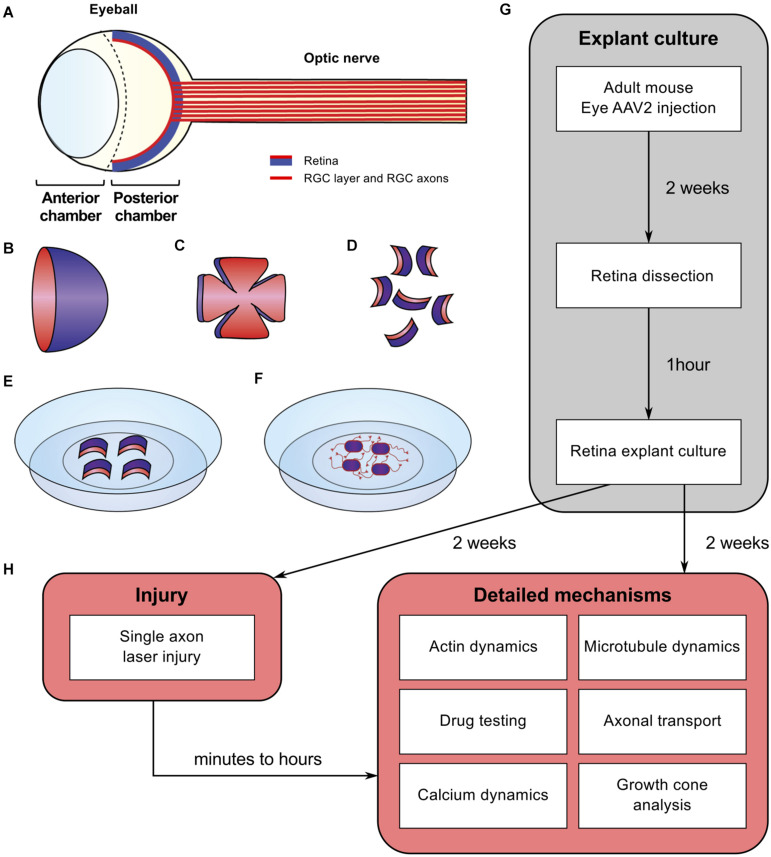
Schematic of experimental procedure for adult retina explant cultures and examples of *ex vivo* analyses. **(A)** Schematic of the eye. The retina is covering the inner side of the eye ball posterior chamber. Retinal ganglion cells are located in the anterior face of the retina (in red) and sending their axons to form the optic nerve **(B)** The retina dissected out of the eye ball is cup-shaped. The RGC layer (in red) is located in the inner side of the cup. **(C)** The retina is cut in flower shape. **(D)** The retina is chopped into small pieces (500 μm in diameter). All the pieces will show a curvature. The RGC layer (in red) is on the concave side. **(E)** Put the RGC side (in red) onto the coverslip coated with poly-L-lysine and laminin and covered with a thin layer of coating medium. **(F)** Schematic of an adult retina explant culture with axons growing on the substrate from RGC. **(G)** Timeline of standard experiment. **(H)** Examples of downstream applications, including *ex vivo* single axon laser injury and/or *ex vivo* analysis of axon biology (e.g., cytoskeleton dynamics, growth cone dynamics, axonal transport).

Here, we propose a method to translate the *in vivo* phenotype into an *ex vivo* approach in order to decipher the molecular and cellular mechanisms underlying mature CNS regeneration. We use adult mouse retina explant in culture, an optimized technique that combines the simplicity of embryonic neuronal cultures with all the characteristics of an adult system. These cultures allow us also to study growth cone behavior, axon guidance modalities, organelle and cytoskeleton dynamics at a single axon level and in response to injury. Similarly, to embryonic neuronal cultures, the adult retina explant system is an evolutive toolbox to test several cellular functions and to study the fine mechanisms of axon growth (Figure 1). Our model is a valuable tool to address all the current questions regarding physiological and pathological events that are difficult to study *in vivo*.

## Animals, Material and Equipment

### Animals

Animal care and procedures were performed according to theGrenoble Institut Neurosciences, French and European guidelines. We used PTEN^*fl/fl*^/YFP-17, PTEN^*fl/fl*^ and PTEN^*fl/fl*^/SOCS3^*fl/fl*^ mice lines in this study, regardless of their sex, aged at least 4 weeks.

### Anesthetic

Ketamine (Clorkétam 1000, Vetoquinol)
Xylazine (Rompun 2%, BAYER)
NaCl 0.9% (VWR, AGUE64812).

### Surgery and Dissection Material

1 mini Bulldog serrefines clamp (Fine Science Tools, 18053-28)
1 pair of laminectomy forceps Dumont #2 (Fine Science Tools, 11223-20)
2 pairs of forceps Dumont #5 (Fine Science Tools, 11251-20)
1 pair of spring scissors (Fine Science Tools, 91500-09)
Surgical scalpel blades no. 11 (Swann-Morton, 0203)
Scalpel handle no. 3 (Fine Science Tools, 91003-12)
50 μl syringe (Hamilton, 80521)
22 gauge tubing
Capillaries (Sutter Instruments, BF150-86-10): pull glass tubes with a needle puller (Sutter Instruments, Flaming/Brown micropipette puller model P-97) with following parameters: heat = 510, pull = 55, vel = 75, time = 120.

### Culture

Glass-bottom dish (MatTek, P35G-1.0-20-C)
Glass coverslips 25 mm (VWR, 631-0172P)
Glass coverslips 12 mm (Marienfeld, 0111520)
35 mm cell culture dishes (Corning, 353001)
4-well culture dishes (Thermo Fisher Scientific, 176740)
Syringe filters 0.2 μm (VWR, 28143-936)
Poly-L-lysine (Sigma-Aldrich, P1399)
Laminin (Sigma-Aldrich, L2020)
Neurobasal-A culture media (Thermo Fisher Scientific, 10888022)
Hibernate A (BrainBits, HA)
Hibernate A minus Calcium (BrainBits, HACA)
Methyl Cellulose (Sigma-Aldrich, M0512)
B-27 supplement (Thermo Fisher Scientific, 12587-010)
L-Glutamine (Corning, 25-005-CI)
Penicillin/streptomycin 10,000 U/ml (Thermo Fisher Scientific, 15140122)
Filtered ultrapure water.

### Chemicals and Other Reagents

Phosphate-buffered saline (PBS) (Euromedex, ET330-A, 10x solution - prepare as a 1x solution by diluting in ultrapure water)
Dulbecco’s PBS (DPBS) 1x without calcium and magnesium (Corning, 21-031-CVR)
Paraformaldehyde (PFA) (Sigma-Aldrich, 441244)
Sucrose (Sigma-Aldrich, S9378)
Filter paper (Dutscher, 074021)
Triton X-100 (Sigma-Aldrich, T8787)
Bovine serum albumin (BSA) 30% (Sigma-Aldrich, A9576)
Primary antibodies: anti-β Tubulin III (TUJ1) (Covance, MMS-435P; Abcam, ab18207), anti-Tau (Millipore, MAB3420), anti GFP (Abcam, ab13970), anti-RBPMS (Millipore, ABN1376)
Secondary antibodies: Alexa Fluor-conjugated secondary antibodies (Jackson ImmunoResearch)
Tetramethylrhodamine B isothiocyanate (TRITC)-conjugated phalloidin (Sigma-Aldrich, P1951)
Fluoromount-G with DAPI (Thermo Fisher Scientific, 00-4959-52)
MitoTracker Red CMXRos (Thermo Fisher Scientific, M7512)
LysoTracker Red DND-99 (Thermo Fisher Scientific, L7528)
Cholera toxin subunit B (CTB), Alexa Fluor 555-conjugated (Thermo Fisher Scientific, C22843)
Absolute ethanol (VWR Chemicals, 20821.365)
Hexane (Sigma-Aldrich, 296090)
Benzyl benzoate (Sigma-Aldrich, B6630)
Benzyl alcohol (Sigma-Aldrich, 305197)
Tissue freezing medium (MM-France, F/TFM-C)
Adhesion slides SuperFrost Plus (Thermo Fisher Scientific, J1800AMNZ).

### Equipments and Softwares

Cryostat device (e.g., Thermo Fisher Scientific, CryoStar NX50 Cryostat)
Platform shaker
Magnetic stirrer with heating
Epifluorescence microscope (e.g., Axio scan slide scanner Scan.Z1, Zeiss, with ZEN 2 slide scan application for automatic stitching)
Confocal microscope (e.g., Andor Dragonfly spinning disk confocal microscope, Oxford Instruments)
Live cell imaging culture chamber equilibrated at 37°C and 5% CO_2_
Laser ablation device (e.g., Andor MicroPoint, Oxford Instruments)
Imaging and analysis software (e.g., MetaMorph)
Image processing software (e.g., ImageJ version 2.00-rc-69/1.52p, with Neurite-J plugin for axonal growth and KymoToolBox plugin for kymograph construction and analysis)
Statistical analysis software (e.g., GraphPad Prism version 7.00).

## Procedures for *ex vivo* Analysis of Retina Explant Cultures

### Solutions to Prepare in Advance

#### Poly-L-lysine Stock Solution - Timing: 15 min

Reconstitute as 5 mg/ml in ultrapure water. Filter with 0.2 μm filter and keep aliquots at −20°C.

#### Coating Medium - Timing: Several Hours

Dissolve 0.2 g of Methyl cellulose in 25 ml of Hibernate A at 4°C using end-over-end shaking (can take several hours). Add 1 ml of B-27 and 500 μl of L-glutamine and complete to 50 ml with Hibernate A. Filter through a 0.2 μm filter. Make 1 ml aliquots and store them at −20°C (up to 6 months).

#### Fixative (8% Formaldehyde 3% Sucrose) - Timing: Several Hours

Dissolve 40 g paraformaldehyde in 400 ml PBS by stirring and heating to 65°C (under chemical hood). Cool down to room temperature. Add 15 g sucrose and dissolve by stirring at room temperature. Complete to 500 ml with PBS. Filter through a filter paper and make 10 ml aliquots. Store at −20°C.

### *In vivo* Procedures

#### Intravitreal Viral Vector Injection (2 Weeks Before Culture)

Intravitreal injections are performed as described before (Park et al., 2008). Anesthetize 4-week-old animals with intraperitoneal injection of ketamine (60–100 mg/kg) and xylazine (5–10 mg/kg). Clamp the external edge of the eye using a mini bulldog serrefines clamp to display the conjunctiva. Using a glass micropipette connected to a Hamilton syringe, inject 1 μl of adeno-associated virus type (AAV2) (at least 10^11 viral particles per ml) into the vitreous body. Viral vectors used in this study are AAV2-Cre, AAV2- CNTF, AAV2-c-myc, AAV2-MitoDsRed or AAV2-Lifeact-tdTomato. Mice with eye inflammation or damage are excluded from the rest of the experiments.

### Retina Explant Culture

#### Coat Glass Coverslips or Glass-Bottom Dishes (One Day Before Culture) - Timing: 15 min on Day 1, 2 h 30 min on Day 2

Cover glass coverslips or glass-bottom dishes (MatTek) with poly-L-lysine (0.5 mg/ml in ultrapure water) and leave it overnight at room temperature. Wash glass with ultrapure water and cover with laminin (20 μg/ml) for at least 2 h at room temperature. Wash twice with ultrapure water and keep in Neurobasal-A until explants are ready.

#### Prepare Culture Medium (On the Day of Culture)

Composition of culture medium: Neurobasal-A, 2% B-27, 20 mM L-glutamine and 5000 units of penicillin/streptomycin. On the day of the culture, mix 1 ml of B-27, 500 μl L-glutamine and 500 μl of penicillin/streptomycin. Complete to 50 ml with Neurobasal-A. Keep at room temperature until use. Store at 4°C (up to one week).

#### Retina Explant Culture - Timing: 30 min per Animal

The procedure is described in Figure 1. Two weeks after viral vector injection, sacrifice animals by cervical dislocation following the institution’s guidelines. Remove eyes quickly using Dumont’s forceps #5 and put in ice-cold Neurobasal-A medium without calcium and magnesium. Under a dissection scope, open eyeball and cut along the line between the anterior and posterior chambers of the eye using spring scissors. The retina is attached in the inferior part of the eye ball. Dissect retina out using forceps #5 and place it in a new dish containing ice-cold Hibernate A medium without calcium and magnesium. Using a scalpel, cut retina into small pieces (about 500 μm in diameter). Other instruments such as punch needles (Gaublomme et al., 2013; Buyens et al., 2014) and tissue chopper (Doussau et al., 2017) could be used to cut retina. In our study, we found that cutting pieces with scalpel blade is the most convenient to set up *ex vivo* cultures and the most reproducible in terms of explant size. Each retina explant presents a slight curvature. The layer of RGC is on the concave side of the explant. Remove Neurobasal-A from coverslips or glass-bottom dishes previously coated with poly-L-lysine and laminin. Lay a thin layer of coating medium and place retina explants with RGC layer facing down (on the coated side of glass coverslip or glass-bottom dish). Put up to two explants per small coverslip, up to four explants per large coverslip, or per glass-bottom dish. After 5 min at room temperature, add culture medium gently. At this step, all explants should adhere to the glass coverslip or glass-bottom dish. If they detach, explants should be removed from the culture. Keep cultures at 37°C 5% CO_2_ for up to 2 weeks.

### Analysis of Fixed Explant Cultures

#### Immunostaining (2 Weeks After Culture) - Timing: 5 h

All steps are carried out at room temperature. Fix cultures by adding gently the same volume of fixative (8% formaldehyde 3% sucrose) as the volume of medium directly in culture dish. Incubate 15 min. Wash three times 10 min with PBS. Permeabilize cultures by incubating in PBS 0.1% Triton X-100 (Sigma Aldrich) for 10 min. Incubate cultures with primary antibodies for 2 h diluted in blocking solution (3% BSA in PBS) (e.g., anti-β Tubulin III (TUJ1) 1:400, anti-Tau 1:250 and/or anti GFP 1:500). Wash three times 10 min with PBS. Incubate cultures with secondary antibodies for 1h diluted in blocking solution (e.g., Alexa-conjugated antibodies 1:500 and/or TRITC-conjugated phalloidin 1:400 for actin staining). Wash three times 10 min with PBS and mount using Fluoromount-G.

#### Explant Axon Outgrowth Quantification

Image explants with epifluorescence microscopy with automatic stitching (e.g., Axio scan slide scanner Scan.Z1, Zeiss, or Fiji image processing). In our study, explants with no or little axon outgrowth (0 or 1 axon) were removed and analyzed separately (Table 1). For the rest of the explants, axon outgrowth can be quantified with a Sholl analysis using the ImageJ plug-in Neurite-J (Torres-Espín et al., 2014). Definition of the explant and background noise filtering can be performed automatically, with manual correction if necessary, as described by the plug-in developer. In our study, the number of neurites intersects was determined by the Sholl analysis with a step of 50 μm. Data are plotted as the number of neurite intersects as a function of the distance to the explant border.

**TABLE 1:** Quantification of axon growth on adult retina explants.

Condition	Total number explants	Number explants with no axon growth	Number explants for Neurite-J quantification^1,2^
PTEN^*fl/fl*^ + AAV2-Plap	47	18 (38.3%)	29 (61,7%)
PTEN^*fl/fl*^ + AAV2-Cre	41	8 (19.5%)	33 (80,5%)
PTEN^*fl/fl*^/SOCS3^*fl/fl*^ + AAV2-Cre + AAV2-CNTF + AAV2-c-myc	64	6 (9.4%)	22 (90,6%)

*^1^Explants with no axon growth were excluded from quantification. ^2^Explants with overlap of axons from neighboring explants were excluded from quantification.*

### Live Imaging of Explant Cultures

#### Live Imaging (2 Weeks After Culture) - Timing: up to 2 h per Axon

In this section, imaging rates and imaging times can be adapted depending on specific mechanism of interest. In our study, all live imaging and laser ablation experiments were performed with PTEN^–/–^ retina explant cultures or PTEN^–/–^/Thy1-YFP retina explant cultures. For fluorescence live imaging, replace culture medium with unsupplemented Hibernate-A with no phenol red. Leave retina explant cultures to equilibrate in the live cell imaging chamber at 37°C and 5% CO_2_ for 15 min before live imaging with or without laser ablation. Image axons or growth cones with confocal microscopy (e.g., DragonFly spinning disk confocal microscope from Andor, controlled with MetaMorph imaging software), either with fluorescence or with DIC illumination, at a rate of 1 image per second or 1 image every 2 s. To work at single axon level and avoid fasciculated axons, choose axons that are isolated from their neighbors. Check axon health by observing and recording growth cone dynamics for 20 min before starting the experiment.

#### Single Axon Laser Ablation

Perform laser ablation, e.g., with Micropoint controlled with MetaMorph imaging software. Calibrate the galvo positions of the Micropoint before each experiment to ensure accurate targeting of the axon. Monitor the cut visually with DIC illumination and recording. In our study, laser ablation settings were: number of pulses set to 4; attenuation plate set to 25% transmission, with possible increase to up to 70%. Do not perform more than two attempts of laser ablation to avoid rapid and irreversible degeneration of the axon. We recorded growth cones and/or axons for 20 min before laser ablation, then 1 h after laser ablation, with 1 image every 2 s. Laser ablation was performed at a distance of about 100 μm from the growth cone. For organelle tracking, laser ablation was performed at a distance of 100 to 200 μm from the growth cone in a region where the axon was straight enough to facilitate definition of region of interest in downstream kymograph analysis.

#### Organelle Live Tracking

Replace culture medium with unsupplemented Hibernate-A with no phenol red. Incubate retina explant cultures with a live tracking dye, e.g., MitoTracker for 5 min (0.1 μM final concentration), or with LysoTracker for 30 min (0.1 μM final concentration). In our study, we recorded fluorescence in single axons 5 min before laser ablation, then 5 min after laser ablation, with one image every second. Up to three axons of the same explant could be recorded.

### Analysis of Live Explant Cultures

#### Organelle Live Tracking Quantification

Quantification of organelle (e.g., mitochondria or lysosomes) dynamics can be performed with imaging software, e.g., the Fiji plug-in KymoToolBox (Zala et al., 2013). In our study, the region of interest was defined as a segmented line along the axon in the proximal part, about 15 μm from the laser ablation point and of about 120 μm in length (Figure 5C). Kymographs were automatically drawn using the Draw Kymo command. A total of 9 axons were analyzed for each organelle. For kymograph analysis, 9 to 18 lysosomes and 13 to 25 mitochondria were selected and the trajectories manually drawn with segmented lines. Kymographs were analyzed with the Analyze Kymo command with the following parameter: minimum speed = 0.02 μm/s. Calculate kinetics parameters according to Virlogeux et al. (2018), as following: anterograde velocity = Vma (μm/s) = Anterograde Distance (μm) / Time (s), retrograde velocity = Vmr (μm/s) = Retrograde Distance (μm) / Time (s), pausing time = average(pausing time per axon), linear flow rate = Q (μm/s) = | Vma| × Number of anterograde organelles + | Vmr| × Number of retrograde organelles.

### Statistical Analysis

Perform statistical analysis with a software for statistical analysis, e.g., GraphPad Prism (version 7.00). In our study, the Shapiro–Wilk test was used to assess normal distribution of each dataset (*P*-value ≥ 0.01). Datasets normally distributed were analyzed with an unpaired Student’s *t*-test (for comparison of two conditions) or an ANOVA test (for multiple comparisons). Datasets that were not normally distributed were analyzed with a Mann-Whitney test (for comparison of two conditions) or a Kruskal-Wallis test (for multiple comparisons).

## Procedures for *in vivo* Analysis of Axon Regeneration

### Solutions to Prepare in Advance

#### Fixative (4% Formaldehyde) - Timing: Several Hours

Dissolve 40 g in 1l of PBS by stirring and heating at 65°C (under chemical hood). Cool down on ice and filter through a filter paper. Keep at 4°C for up to one week.

#### Cryoprotectant (15% Sucrose) - Timing: 1 h

Dissolve 7.5 g sucrose in 40 ml PBS by mixing end-over-end at room temperature (can take up to 1 h). Complete to 50 ml with PBS and keep at 4°C for up to one week.

### *In vivo* Procedures

#### Intravitreal Viral Vector Injection (4 Weeks Before Termination)

Intravitreal injections are performed as described above.

#### Optic Nerve Crush (2 Weeks Before Termination)

Two weeks after viral injection, perform optic nerve crush. Anesthetize 6-week-old animals with intraperitoneal injection of ketamine (60–100 mg/kg) and xylazine (5–10 mg/kg). Open the conjunctiva with fine scissors. Carefully slide dilating forceps in-between the two arteries behind the eye ball to expose the optic nerve underneath. Pinch the optic nerve for 5 s using Dumont #5 forceps 1 to 2 mm behind the eye ball. Animals with unstoppable heavy bleeding are excluded from the study.

#### Intravitreal CTB Injection (2 Days Before Termination)

Following the same procedure as intravitreal viral vector injection, and 2 days before termination, inject 1 μL of Alexa 555-conjugated CTB at 1 μg/μL into the vitreous body of the eye.

### Optic Nerve and Retina Processing

#### Eye and Optic Nerve Dissection

Perfuse mice intracardially with ice-cold PBS for 3 min, then with ice-cold 4% formaldehyde in PBS for 3 min. Dissect out the eye balls and the optic nerves. Post-fix samples overnight at 4°C in 4% formaldehyde. Separate eye balls and optic nerves carefully with fine scissors.

#### Whole Optic Nerve Clarification – Timing: 1 h 30 min on Day 1, 2 h 30 min on Day 2

Clarification procedure is adapted from Dodt et al. (2007). All steps are carried out at room temperature unless specified otherwise. After post-fixation, wash optic nerves three times 10 min in DPBS without calcium and magnesium. Dehydrate optic nerves progressively in ethanol: 20 min in 50% ethanol/DPBS, 20 min in 80% ethanol/DPBS, 20 min in 95% ethanol/DPBS. Incubate in 100% ethanol overnight at 4°C. Incubate optic nerves in hexane for 2 h. Transfer optic nerves in a mix of benzyl benzoate:benzyl alcohol 2:1 and let optic nerves clarify for about 10 min. Keep in the dark at 4°C until imaging.

#### Eye Ball Cryosectioning – Timing: 2 Days Cryopreservation, 4 h Freezing, 1 h Cryosectioning Per Mold

After post-fixation, wash eye balls three times 10 min in PBS. Dehydrate eye balls in 15% sucrose for at least 48 h at 4°C. Embed eye balls in tissue freezing medium in embedding molds, with 3 to 5 eyes of the same condition in one mold. Freeze molds at −80°C for at least 4 h. Perform sagittal cryosectioning, setting section thickness to 14 μm and mounting sections on superfrost slides. Sections can be kept at −20°C until immunostaining.

#### Retina Immunostaining – Timing: 1 h 30 min on Day 1, 3 h on Day 2

Defrost slides at room temperature for 20 min. All steps are carried out at room temperature unless specified otherwise. Wash slides three times 10 min in PBS. Incubate slides in blocking solution (PBS 0.1% Triton X-100 3% BSA) for 1 h. Incubate samples with primary antibodies diluted in blocking solution overnight at 4°C (e.g., anti-RBPMS 1:250, anti-GFP 1:500). Wash slides three times 10 min in PBS. Incubate slides in secondary antibodies diluted in blocking solution for 2 h (e.g., Alexa-conjugated antibodies 1:500). Wash three times 10 min with PBS and mount using Fluoromount-G.

### Imaging and Analysis of Axon Regeneration

#### Axon Regeneration Imaging

For whole optic nerve imaging, image optic nerve with confocal microscopy, e.g., the DragonFly spinning disk confocal from Andor. Acquire z stacks setting each z step to 2 μm to scan the entire width of the cleared optic nerves. In our study, we used a custom stitching module in MetaMorph to stitch images with at least 10% overlap. Perform the maximum z projection of stacks to visualize and quantify axon regeneration.

#### Axon Regeneration Quantification

The principle is described in Supplementary Figure S1. Use an image processing software for quantifications, e.g., ImageJ. Proceed with the maximum projection of the z-stack acquisition of transparent optic nerves (16-bit image). Define the injury site manually with a straight line as the site where CTB labeling drops in intensity in the optic nerve. Measure the fluorescence intensity profile at specific distances from the injury site (e.g., 200, 500, 750, 1000, 1500, 2000, 2500, and 3000 μm) along a line manually drawn orthogonally to the optic nerve, and with a length corresponding to the optic nerve width. Measure the intensity profile in a region with no regeneration (background measurement). Calculate the integrated fluorescence intensity at each step, e.g., using R, and normalize to the optic nerve width at each step (that may vary along the optic nerve). Normalize the integrated intensity to the maximal intensity value of all steps in the regenerating region to account for variations between optic nerves. Finally, subtract the normalized integrated intensity of background from the normalized integrated intensity at each step. In our study, the results are plotted in arbitrary units as a function of the distance from the injury site.

### Statistical Analysis

Perform statistical analysis as described above.

## Results

### Neurites Are Axons From RGC

**FIGURE 2:**
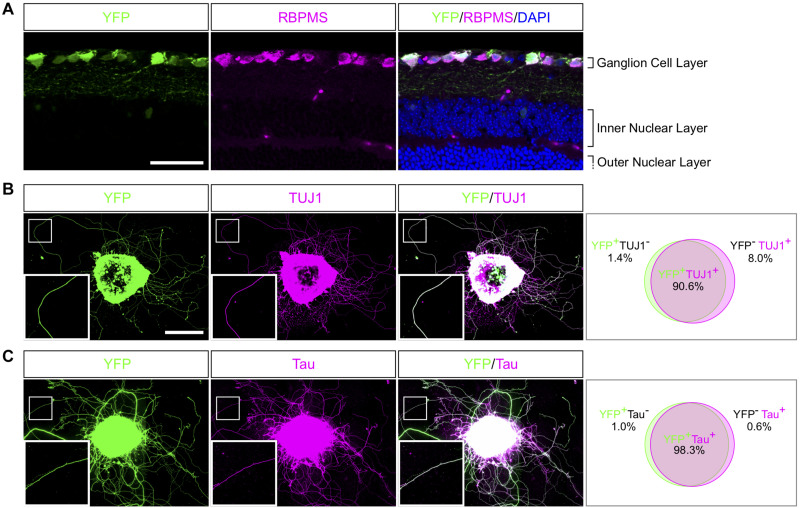
Characterization of neurites in adult retina explant culture. **(A)** Representative confocal picture of a PTEN^*fl/fl*^/Thy1-YFP adult mouse retina section. RGC expressing YFP (labeled in green) are stained with anti-RBPMS (magenta) antibody and nuclei are labeled with DAPI (blue). Scale bar: 50 μm. **(B,C)** Representative pictures of an adult retina explant from a PTEN^*fl/fl*^/Thy1-YFP mouse with *a priori* intravitreal injection of AAV2-Cre. RGC neurites are labeled with YFP (in green), anti-β Tubulin III (TUJ1) (magenta) **(B)** or anti-Tau (magenta) **(C)**, with corresponding quantification of colocalization. At least 350 neurites from at least 18 explants and at least three independent experiments were quantified. Scale bar: 500 μm.

The mechanisms underlying axon regeneration in mature CNS remain difficult to address. While it is common to use embryonic neuronal cultures to address those questions, the intrinsic ability of young neurons to regrow their axon induces a bias regarding the molecular and cellular mechanisms of axon growth in mature neurons (Goldberg et al., 2002). Adult retina and optic nerve are gold-standard models to study neuron survival and axon regeneration in the CNS. Here, we show how to culture retina explants from adult mouse (Figure 1). In this set up, RGC neurons are not dissociated in culture but are kept in a whole retinal structure. Because of the intrinsic growth incompetence of adult CNS neurons, we observed that wild-type (WT) explants grow few neurites (Table 1). Therefore, we activated the mTOR pathway, known to induce axon regeneration in mature CNS (Park et al., 2008), through deletion of PTEN. Using the PTEN^*fl/fl*^/YFP-17 mouse line (Sun et al., 2011), we injected 1 μL of AAV2-Cre into the vitreous body of the eye to delete PTEN in RGC. The AAV2 injection strategy allows to target the majority of RGC (Park et al., 2008) with high specificity, as YFP is mostly expressed by RGC and few amacrine cells (Sun et al., 2011; Figure 2A). Two weeks after injection, we dissected out the retinas and put the adult explants in culture (Figure 1). After 2 weeks in culture, we performed immunocytochemistry using an anti-β Tubulin III (TUJ1) antibody to label neurites. We found that 90.6% of neurites were YFP^+^, meaning that almost all the neurites that grow out of the explant tissue are from RGC (Figure 2B). To address whether these neurites are axons, we used an anti-Tau antibody, a specific axon marker. We found that 98.3% of YFP^+^ neurites are Tau^+^, meaning that the vast majority of the neurites growing out of the explants are RGC axons (Figure 2C). Therefore, this *ex vivo* set up is ideal to study molecular and cellular mechanisms of axon regeneration specifically in mature RGC at a single axon level.

### Adult Retina Explant Cultures Recapitulate *in vivo* Phenotypes

**FIGURE 3:**
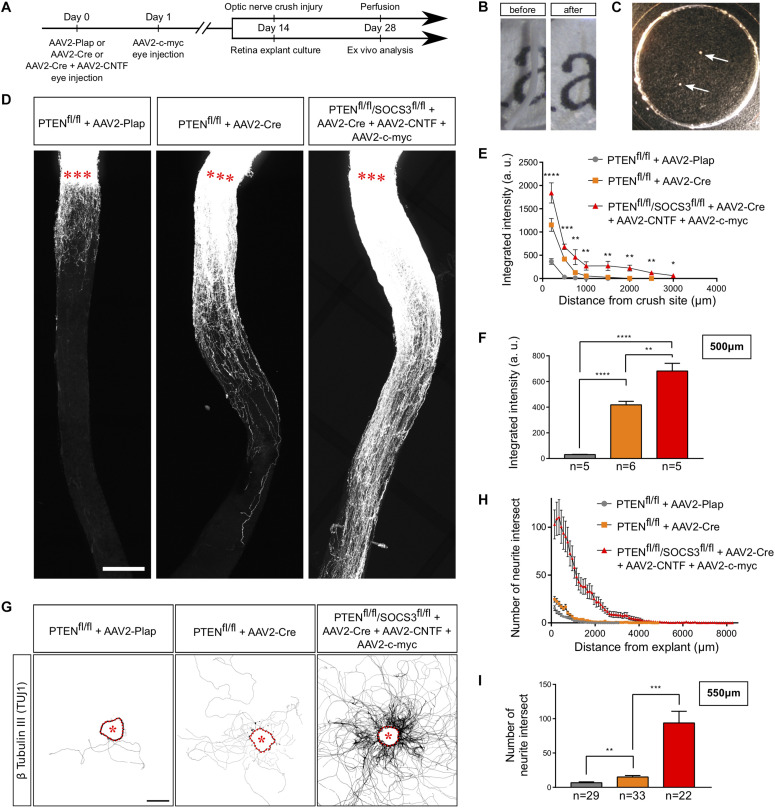
Adult retina explant culture mimics *in vivo* axon regeneration. **(A)** Timeline of experiments. **(B)** Picture of mouse optic nerve before and after transparisation. **(C)** Picture of retina explants (arrows) in culture in a glass-bottom dish. **(D)** Representative confocal pictures of whole optic nerve showing axon regeneration 2 weeks post-injury in control (PTEN^*fl/fl*^ + AAV2-Plap), PTEN-deleted (PTEN^*fl/fl*^ + AAV2-Cre) and PTEN/SOCS3 co-deleted c-myc-overexpressing (PTEN^*fl/fl*^/SOCS3^*fl/fl*^ + AAV2-Cre + AAV2-CNTF + AAV2-c-myc) conditions. Axons are traced with fluorescently-labeled CTB. Red stars indicate the injury site. Scale bar: 200 μm. **(E)** Quantification of axon regeneration (integrated intensity) in panel **(D)**. Data expressed as mean ± s.e.m. ANOVA test. **(F)** Quantification of axon regeneration at 500 μm of injury site. Unpaired *t*-tests. *p < 0.05, **p < 0.01, ***p < 0.001, ****p < 0.0001. **(G)** Representative pictures of adult retina explants after 2 weeks in culture, from control (PTEN^*fl/fl*^ + AAV2-Plap), PTEN-deleted (PTEN^*fl/fl*^ + AAV2-Cre) and PTEN/SOCS3 co-deleted + c-myc-overexpressing (PTEN^*fl/fl*^/SOCS3^*fl/fl*^ + AAV2-Cre + AAV2-CNTF + AAV2-c-myc) conditions. Axons are labeled with anti-β Tubulin III (TUJ1) antibody. The star indicates the explant. Scale bar: 500 μm. **(H)** Quantification of axon growth in panel **(G)** with Sholl analysis. Data expressed as mean ± s.e.m. **(I)** Quantification of axon growth (number of axon intersect) at 550 μm of explant. Mann-Whitney tests. **p < 0.01, ***p < 0.001.

An *ex vivo* system is highly relevant when it recapitulates the phenotype of the corresponding *in vivo* model. It is now largely described that wild-type adult CNS neurons are not able to grow axons after injury and that modulating neurons themselves enable CNS axon regeneration (He and Jin, 2016). Extensive axon regeneration in the mature CNS is triggered by the activation of the mTOR pathway (Park et al., 2008), which leads to axon growth over several hundreds of micrometers from the injury site. Furthermore, long distance regeneration from the eye ball to the brain has been obtained by the synergistic activation of mTOR, JAK/STAT and c-myc pathways in RGC (Belin et al., 2015). Using these established models of CNS regeneration, we compared growth extent *in vivo* in the optic nerve and *ex vivo* in adult retina explants (Table 1 and Figure 3A). To do so, we used PTEN-floxed (PTEN^*fl/fl*^) and PTEN^*fl/fl*^/SOCS3^*fl/fl*^ mouse lines, as PTEN and SOCS3 are negative regulators of mTOR and JAK/STAT pathways, respectively. We injected 1 μL of AAV2-Cre into the vitreous bodies of P28 PTEN^*fl/fl*^ mice to delete specifically PTEN from RGC. AAV2-Cre, AAV2-CNTF (to activate JAK/STAT pathway) and AAV2-c-myc were injected into the vitreous bodies of P28 PTEN^*fl/fl*^/SOCS3^*fl/fl*^ mice. As control, we used PTEN^*fl/fl*^ mice injected with AAV2-Plap. Two weeks after injection, we proceeded with optic nerve injury or with retina explant culture (Figures 3A–C). With this experimental design, we could focus on the same tissue at the same stage for *in vivo* and *ex vivo* experiments.

For the *in vivo* part, we injected Alexa Fluor 555-conjugated cholera toxin B (CTB-555) in the vitreous body 48 h before sacrificing the animals to label regenerative fibers. Optic nerves were subsequently dissected and cleared (Figure 3B), then the whole tissue was imaged with confocal microscopy to assess the extent of axon regeneration. As previously shown (Park et al., 2008), WT axons show very little outgrowth from the injury site, while PTEN^–/–^ axons display robust regeneration (Figures 3D–F). Consistent with what was achieved previously (Belin et al., 2015), the co-activation model PTEN^–/–^/SOCS3^–/–^+c-myc resulted in long-distance robust regeneration after 2 weeks (Figures 3D–F and Supplementary Figures S1A–C). We set up a semi-automated method using Image J software (Supplementary Figure S1D) to quantify the extent of regeneration without any bias.

In parallel, after 2 weeks in culture, explants were fixed and stained with an anti-β Tubulin III (TUJ1) antibody. In WT conditions, 38% of all explants did not grow any axon, compared to 19.5% for PTEN^–/–^ and less than 10% for PTEN^–/–^/SOCS3^–/–^+c-myc conditions (Table 1). This observation reflects *in vivo* conditions as WT optic nerves show very little regeneration compared to PTEN^–/–^ and PTEN^–/–^/SOCS3^–/–^+c-myc conditions. For the explants that grew more than one axon, we measured axon number and length in WT, PTEN^–/–^ and PTEN^–/–^/SOCS3^–/–^+c-myc conditions (Figure 3G–I and Supplementary Figures S2A–D). To do so, we used the Sholl analysis-based plug-in Neurite-J (Torres-Espín et al., 2014) on Image J software and measured the number of intersections (outgrowing neurites) at defined distances of the explants (50 μm step) (Supplementary Figure 2E). This method allows to quantify growth extent (axon number and length) without any bias of manual counting. As expected, neurite outgrowth was very weak in control conditions, as only few axons came out of the explant and with a very short outgrowth (Figures 3G–I and Supplementary Figure S2). PTEN^–/–^ explants showed an intermediate phenotype like in the *in vivo* condition. In contrast, for PTEN^–/–^/SOCS3^–/–^+c-myc explants, a very high number of axons grew far from the explant border (up to 8 mm) (Figures 3G–I and Supplementary Figure S2).

Altogether, our experiments show that the culture system doesn’t impair the capacity of adult axons to respond to activation of pro-regenerative molecular pathways. Importantly, this method allows us to isolate the retina and recreate in culture the growth conditions for adult axons that mimic *in vivo* features. Therefore, it is a relevant model to study regeneration at a single-axon level, which so far is not doable *in vivo* in the adult mammalian CNS.

### Laser Guided Axon Ablation to Mimic CNS Lesion in a Dish

**FIGURE 4:**
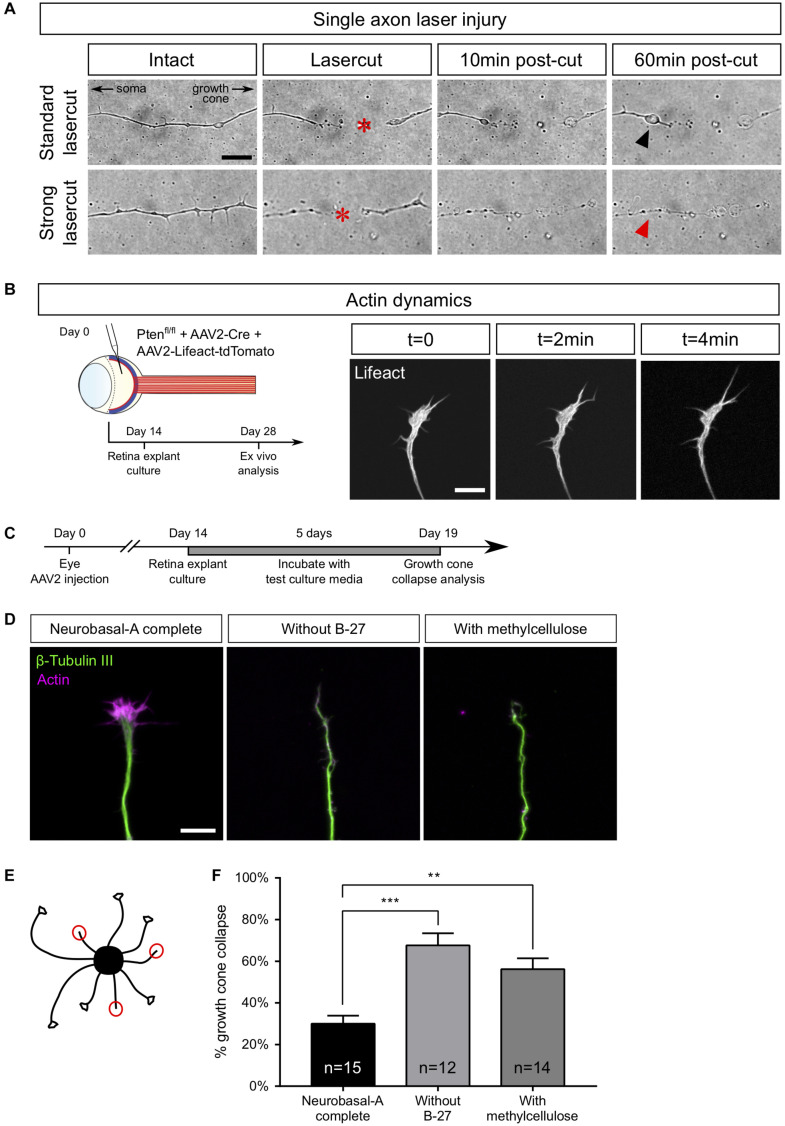
Examples of *ex vivo* analysis in adult retina explant cultures. **(A)** Single axon laser injury performed with MicroPoint Laser Illumination (Andor, Oxford Instrument). After lasercut (red star), a retraction bulb (black arrowhead) forms in the proximal part of the axon, while the distal part degenerates. In the case of a high-power (strong) lasercut, both proximal (red arrowhead) and distal parts degenerate. **(B)** Intravitreal injection of AAV2-Lifeact-tdTomato allows live visualization of actin cytoskeleton dynamics in a growth cone of adult retina explant culture. **(C)** Timeline of experiment of growth cone collapse assay in PTEN^–/–^/SOCS3^–/–^+ c-myc adult retina explant cultures. **(D)** Representative confocal pictures of growth cones of adult retina explant cultures in standard culture medium (Neurobasal-A complete), without B-27 and with methylcellulose. Scale bar: 10 μm. **(E)** Schematic drawing showing the analysis of a collapse assay. Collapsed growth cones are indicated in red circles. **(F)** Quantification of growth cone collapse. Data expressed as means ± s.e.m. Kruskal-Wallis test with comparison to control condition (Neurobasal-A complete). **p < 0.01, ***p < 0.001.

Using our *ex vivo* retina set up, we next studied axon injury at a single axon level (Figure 4A). We used laser ablation of single axons, in a similar approach to what is described *in vivo* in Drosophila or C. Elegans (Byrne et al., 2011; Soares et al., 2014) or *in vitro* in primary cultures of dissociated mouse embryonic neurons (Difato et al., 2011; Koseki et al., 2017). This technique allows the ablation of an axon without damaging the cell. It enables to study early events that occur after axon injury such as calcium dynamics or later events such as growth cone formation or guidance mechanisms (Figure 1H). We selected a single axon and imaged the growth cone for 20 min (1 image every 2 s) to ensure viability (Supplementary Video S1). Because of the variability in axon length, we chose to define the lesion site between 100 and 200 μm from the tip of the growth cone. Live imaging was performed up to 1 h after the lesion (1 image every 2 s). In some cases, we observed that laser ablation was not efficient to cut the axon. Several scenarii are possible. In most cases, the laser is not aligned with the axon plane. Thus, the cut was effective by adjusting the focus. In other cases, laser power was not sufficient, depending on the thickness of the axon. We increased the attenuation plate (up to 70% transmission) to perform effective ablation (see section “Procedures for *ex vivo* Analysis of Retina Explant Cultures”). However, in order to avoid any unnecessary damage to the axon, we restrained the laser ablation to two attempts per axon. If the cut was incomplete or unsuccessful (in less than 10% cases), we worked with another axon. For acquisitions that lasted over an hour, we studied one axon per explant.

Upon efficient laser ablation, the distal part of the axon undergoes degeneration, as described *in vivo* (Ertürk et al., 2007). Regarding the proximal part of the axon, there were two main outcomes after laser ablation: either a retraction or a fast degradation. In the first case, we observed axon retraction from the lesion site (Figure 4A and Supplementary Video S2). Interestingly, this observation has also been reported *in vivo* (Tom et al., 2004; Ertürk et al., 2007), especially in the case of spinal cord injuries. Thus, *ex vivo* adult retina explant cultures recapitulate the very first events following axonal lesion. In the second case, the degeneration spread fast all along the axon (Figure 4A and Supplementary Video S3). In our *ex vivo* set up, this phenotype meant that the ablation power was too high and damaged the cell. Those axons were excluded from experiments.

### Adult Retina Explant Cultures as Useful Tool to Study Molecular and Cellular Mechanisms of CNS Regeneration: A Few Examples of Application

Axon regeneration relies on several processes, such as growth cone formation, organelle movement or calcium dynamics. We used several experimental set-ups as proof of concept to show that adult retina explant cultures are the ideal tool to address these questions. The list of experiments is not exhaustive and our system can be implemented into many experimental designs to respond to future questioning in the field.

#### Growth Cone Dynamics

The growth cone is a highly motile structure at the end of growing axons. It is essentially composed of a central domain of microtubule and the periphery is formed by actin cytoskeleton (Coles and Bradke, 2015). The growth cone is involved in axon elongation and guidance (Dent et al., 2011; Bradke et al., 2012) and acts as a target-tracking sensor of the navigating axon (Tessier-Lavigne and Goodman, 1996). Indeed, it expresses surface receptors that integrate environment signals such as guidance factors. When a growth cone comes across a repulsive cue, it will retract and change its direction of growth (Dent et al., 2011). It is well admitted that the early sign of repulsion is the collapse of the growth cone (Fawcett, 1993). This drastic change in the growth cone morphology is due to the depolymerization of the cytoskeleton, mainly actin (Bradke et al., 2012).

In the mature CNS, lesioned axons fail to form a growth cone, and growth cone formation is considered as a limiting step in CNS regeneration. Here, we could study growth cone dynamics in live cultures. To do so, we injected AAV2-Lifeact coupled with tdTomato into the vitreous body of PTEN^*fl/fl*^ mice together with AAV2-Cre (Figure 4B). As Lifeact is a small probe that binds specifically to polymerized actin, we were able to record growth cone dynamics as well as actin dynamics in live cultures (Supplementary Video S4).

Growth cones could also be analyzed in fixed cultures to address the question of mature axon response to environmental cues. This is of particular relevance in long-distance regeneration models that elicit guidance defects of regenerating fibers (Luo et al., 2013; Pernet et al., 2013a). For this reason, we used Pten^–/–^/SOCS3^–/–^+c-myc explants and we showed that growth cones elicit a specific collapse response that depends on culture medium composition. We tested several culture conditions, usually used for embryonic cultures, to find the best medium composition to minimize the baseline of growth cone collapse rate in adult retina explant cultures. We incubated explants either in complete (B-27-containing) medium, or in medium without B-27, or in medium supplemented with methylcellulose (Figure 4C). In this case, we fixed cultures after 5 days because at this time, Pten^–/–^/SOCS3^–/–^+c-myc explants display sufficient outgrowth with axons isolated enough one from the other to assess growth cone morphology. Following fixation, we stained with anti-β Tubulin III (TUJ1) antibody and phalloidin, which labels polymerized F-actin (Figure 4D). For each explant, we counted the number of axons exhibiting a non-collapsed (spread) and collapsed (less than 2 filopodia and no lamellipodia) phenotype (Figure 4E). With the complete medium, the basal rate of growth cone collapse was about 30%, which is slightly higher than embryonic cultures of cortical neurons [around 20% (Bechara et al., 2008)]. In contrast, unlike young neurons (Sünwoldt et al., 2017), mature RGC axons are very sensitive to B-27 deprivation. Moreover, replacing B-27 by methylcellulose did not improve growth cone collapse rate, as we found that in both conditions the basal rate was more than 60% (Figure 4F). It is important to note that these media did not prevent axon growth in our cultures, so growth cone dynamics is probably active in the early days of culture. However, it is possible that trophic support of basal medium (B-27-deprived) decreases over time and that supplementing the medium with methylcellulose doesn’t improve the baseline of collapse. We concluded that it is essential to supplement the medium with B-27 to minimize growth cone collapse rate in mature cultures. Determining optimal medium composition opens up the possibility to use adult retina explant cultures to test the repulsive activity of an exogenous guidance cue, as well as the distribution and role of guidance receptor. This provides a relevant tool to understand how adult axons integrate environmental signals, in particular guidance cues. This question is fundamental in CNS axon regeneration because of the lack of guidance or mistargeting of regenerating axons that is observed after injury, for example in the optic chiasm (Luo et al., 2013; Pernet et al., 2013a, b).

#### Axonal Transport

**FIGURE 5:**
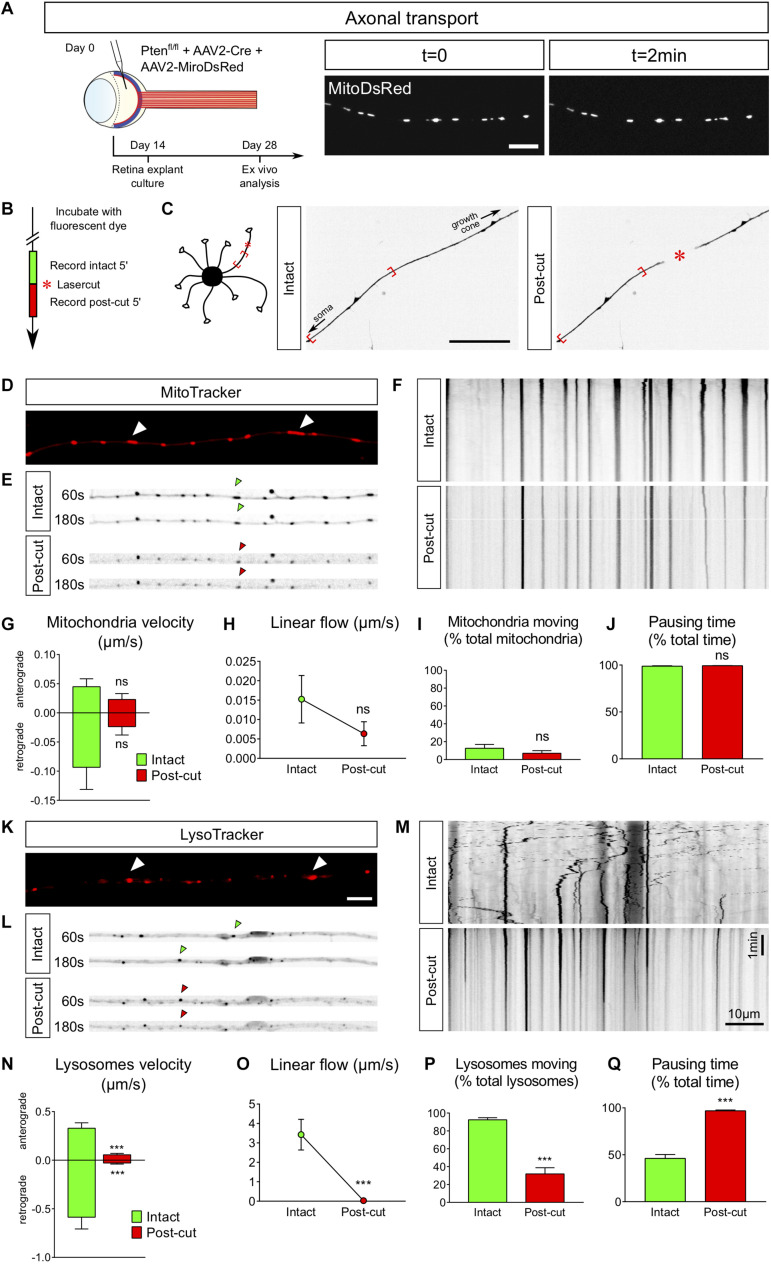
*Ex vivo* analysis of axonal transport in intact versus injured conditions. **(A)** Intravitreal injection of AAV2-MitoDsRed in the eye allows live visualization of mitochondria axonal transport in adult retina explant culture. Scale bar: 10 μm. **(B)** Timeline of live analysis. Adult retina explant cultures are incubated with fluorescent dyes, which allows live tracking of lysosomes or mitochondria. **(C)** Representative pictures of a single axon before and after laser injury (intact and post-cut). The red star indicated the site of laser injury. The red brackets indicate the region recorded for axonal transport analysis. Scale bar: 50 μm. **(D)** Representative confocal picture of MitoTracker Red labeling of an axon, showing mitochondria (white arrowheads). Scale bar: 5 μm. **(E)** Representative pictures of mitochondria transport in an axon in intact and post-cut conditions. Arrowheads show tracking of a single mitochondria over time. **(F)** Representative kymographs of mitochondria tracking in intact and post-cut conditions. **(G–J)** Quantifications of mitochondria velocity, global linear flow, number of moving mitochondria and pausing time. *n* = 9 axons. Mann–Whitney tests. ns: not significant. **(K)** Representative confocal picture of LysoTracker Red labeling of an axon, showing lysosomes (white arrowheads). Scale bar: 5 μm. **(L)** Representative pictures of lysosome transport in an axon in intact and post-cut conditions. Arrowheads show tracking of a single lysosome over time. **(M)** Representative kymographs of lysosome tracking in intact and post-cut conditions. **(N–Q)** Quantifications of lysosome velocity, global linear flow, number of moving lysosomes and pausing time. *n* = 9 axons. Mann–Whitney tests. ***p < 0.001. **(G–J)** and **(N–Q)** Data expressed as means ± s.e.m.

Axons are busy highways for many types of transport. The transport of specific organelles, such as mitochondria, has been shown to be critical to achieve regeneration (Zhou et al., 2016). High frequency moving mitochondria have been linked with axons showing strong growth capability (Cartoni et al., 2017). However, studying mitochondria dynamics *in vivo* remains technically challenging (Takihara et al., 2015). Here we show that adult retina explant cultures are a good tool to address this question. We used two approaches to label mitochondria in live axons: viral infection and incubation with a specific dye. We infected PTEN^*fl/fl*^ adult RGC with AAV2-Cre together with AAV2-MitoDsRed, which labels all mitochondria with DsRed (Figure 5A). In this experimental design, all cells of the explant expressed MitoDsRed, which could be tracked with live confocal imaging (Supplementary Video S5). The second approach is based on the use of live tracking cell-permeant dyes more adapted to study different organelles from the same culture. In this case, we put in culture adult retina explants from PTEN^*fl/fl*^ mice, whose RGC were infected with AAV2-Cre. After 2 weeks, explants were incubated for 5–10 min with MitoTracker, a specific mitochondrion-selective dye that accumulates in active mitochondria; or for 20–30 min with LysoTracker, which labels acidic organelles and enables lysosome tracking. We imaged organelle dynamics 5 min before and 5 min after laser guided axon lesion (Figures 5B,C) and performed a kymograph analysis in the proximal region of the axon close to the lesion site (about 15 μm). Interestingly, we found that in mature intact axons, most mitochondria (90%) are stationary (Figures 5D–J), similar to what was shown *in vivo* (Zhou et al., 2016; Misgeld and Schwarz, 2017). There was only 10% of slowly moving mitochondria. Following laser guided lesion, mitochondria displayed even less movement (in terms of velocity, linear flow and pausing time), although not significantly different from the intact condition where the basal levels are already very low (Figures 5G–I and Supplementary Videos S6A,B).

In contrast to mitochondria in intact condition, we observed that lysosomes, tracked with LysoTracker, were extremely motile (Figures 5K–Q and Supplementary Videos S7A,B), with a bidirectional movement predominantly retrograde, as described for maturing lysosomes tracked with LysoTracker in axons (Gowrishankar et al., 2017). Indeed, 90% of lysosomes were moving (both anterogradely and retrogradely) in intact axons (Figure 5P) with an average retrograde speed of 0.6 μm/s and an average anterograde speed of 0.3 μm/s (Figure 5N). After axon ablation, only 30% of lysosomes kept moving (Figures 5N–P), with an average speed reduced to less than 0.06 μm/s in both directions (Figure 5N). The pausing time increased from 50% in intact conditions to almost 100% after axon injury (Figure 5Q and Supplementary Videos S7A,B). Therefore, axon ablation disrupts lysosome movements in adult axons.

Altogether, our experiments prove that adult retina explant cultures enable to sustain adult axons in culture and to isolate various features critical to achieve axon regeneration in the mature CNS.

## Discussion

### Adult Retina Explants as a Toolbox to Study Axon Regeneration

Understanding CNS regeneration has been a major challenge for centuries since (Ramon y Cajal, 1928) first observations that unlike the PNS, CNS neurons fail to form a growth cone to achieve successful regrowth after lesion. One of the main issues is to decipher the mechanisms underlying mature axon growth. Indeed, it is challenging to sustain adult CNS neurons in culture. Therefore, most studies address the regulation of axon regeneration by using embryonic cultures as an *in vitro* tool. However, this approach presents a major caveat: developing neurons have the intrinsic ability to grow their axon after lesion. The molecular and cellular growth pathways are fundamentally different during development and regeneration. This explains partially why several pathways characterized *in vitro* using such embryonic cultures do not induce extended regeneration *in vivo* (Monsul et al., 2004; Moore et al., 2009; Kurimoto et al., 2010; Heskamp et al., 2013; Pita-Thomas et al., 2019; Thompson et al., 2019).

To circumvent this discrepancy, we propose to use adult retina cultures. Retina explants are relevant because the visual system is part of the CNS and the optic nerve presents the same features as the rest of the CNS regarding injury: RGC axons fail to regenerate and to survive after lesion. Using the optic nerve as a model, the modulation of neuronal intrinsic capabilities has been shown to be key to promote axon regeneration in the mature CNS. Consequently, over the past decade, the optic nerve has become a gold-standard model to address axon regeneration in the CNS. Importantly, axon regeneration is triggered in a similar way in RGC and in other CNS neurons, such as corticospinal neurons or dopaminergic neurons, upon activation of mTOR (Liu et al., 2010; Kim et al., 2011). The clearing approach gives a full phenotypic view of axon regeneration from the eye to the brain with no bias. In parallel, adult retina explant cultures enable to isolate an adult retina in a dish to address the fine molecular and cellular events that control axon regeneration. In combination with phenotypic, high-throughput profiling and functional approaches that can be done mostly *in vivo*, *ex vivo* tools are essential tools to explore axon biology in the context of CNS degeneration and regeneration.

### The *ex vivo* Model: A Window on the Adult Injured CNS

Here, we set up adult retina explant cultures to address the molecular and cellular mechanisms underlying axon regeneration in mature CNS neurons. Explants from PTEN^*fl/fl*^/YFP-17 retina allowed us to show that the vast majority of outgrowing neurites are RGC axons. Thus, the neuronal population of interest (RGC) are able to grow axons in our *ex vivo* set-up. It is now well-understood that the RGC neuronal population is actually heterogenous and composed of more than 40 subtypes so far identified. These RGC subpopulations can be classified according to their anatomy (morphology, dendritic arborization, localization within the retina) and functionality (local network, brain target and physiological activity) (Sanes and Masland, 2015; Baden et al., 2016; Martersteck et al., 2017). In addition, past studies including recent large-scale characterization have allowed to identify molecular markers specific for some subpopulations (Krieger et al., 2017; Rheaume et al., 2018; Berg et al., 2019). Besides, RGC subpopulations do not respond similarly to the injury and do not display the same regeneration potential (Duan et al., 2015; Daniel et al., 2018; Tran et al., 2019). Therefore, it is crucial to understand RGC subpopulation specificity in injury response and regeneration potential. Our *ex vivo* system can be adapted for this purpose by using transgenic mouse lines expressing molecular markers specific for one type of RGC, e.g., KCNG4-YFP mouse reporter line for alpha-RGC (Duan et al., 2014) or OPN4-GFP mouse reporter line for ipRGC (Schmidt et al., 2008). This would allow tracking of axons of specific subpopulations in the adult retina explant culture.

While AAV2 viral vectors mostly target RGC, amacrine cells and a few horizontal cells are also targeted (Sun et al., 2011; Zhang et al., 2019). Specific cell targeting can be achieved by using cell type-specific promoters, such as SNCG for RGC (Chaffiol et al., 2017). In addition, optic nerve injury induces molecular changes in RGC local network, and it was recently shown that RGC non-autonomous modulation of other cell types, such as amacrine cells, can promote RGC regeneration (Zhang et al., 2019). Therefore, our system can be adapted to target specifically other cell types, for instance by using cell type-specific promoters, and study the impact on RGC axon growth *ex vivo*. Furthermore, the main focus of our present study is to unravel and modulate the intrinsic regenerative capabilities of adult CNS neurons. However, the role of the extracellular environment is also of primary importance regarding regeneration. Adult regenerating neurons lie within a complex environment where extracellular matrix, neighboring glial cells and injury-related processes such as inflammation may influence their regenerative potential. One possibility is to address these questions by expanding our *ex vivo* model to cultures on substrate formed of glial cells, as it was done for embryonic cultures (Kleitman et al., 1988), or to cultures on various ECM coating that may influence axon growth.

The timeline of experiments *in vivo* and *ex vivo* is exactly the same, so neurons are studied exactly at the same stage of maturation and gene manipulation *in vivo* and *ex vivo*. In addition, we showed that the retina explant cultures display the same extent of growth as what is observed *in vivo*. This result suggests that the functional mechanisms underlying axon growth are maintained in the *ex vivo* system. In other words, adult axons in culture respond identically to molecular manipulation *in vivo* and *ex vivo*. This essential feature confirms the relevance of using adult retina explant cultures over embryonic neuronal cultures. In this context, the Sholl analysis is relevant for the purpose of assessing axonal length and density of *ex vivo* explants. Based on immunofluorescence on fixed adult retina explant cultures, image analysis tools can be used to reconstruct explant network and quantify various aspects related to axon outgrowth, including total network length, directionality and trajectories of single axons, complexity of dendritic tree and branching, etc. (Arganda-Carreras et al., 2010; Mh et al., 2011; O’Neill et al., 2015).

### Laser Guided Injury of Single Adult CNS Axons *ex vivo*

Our model of adult retina explants allows to recreate the context of adult CNS axon growth in a dish. As an *ex vivo* approach, one limitation is that RGC axons undergo an injury when the retina is dissected out of the eyeball. Our cultures were left for 14 days *in vitro*. At this stage, we considered axons to be in basal (intact) condition, allowing us to focus on the subcellular events that follow laser guided axonal lesion. This strategy has already been used in previous work to study growth cone formation following axotomy (Nawabi et al., 2015). In this study, we describe the optimal parameters of our set-up that led to efficient single axon cut with no global degeneration of the axon (in the proximal region). This is essential to track fine cellular and molecular events in response to the lesion with no technical bias. We present here several downstream applications of interest to the field of degeneration and regeneration. Yet, our *ex vivo* model is not restricted to these examples and can be considered as an evolutive toolbox where conditions (time of culture, position of the laser cut, etc.) should be optimized.

Adult retina explant cultures enable to decipher regeneration mechanisms at the level of single axons. One approach that we described here in PTEN^–/–^ cultures is to perform laser guided ablation of a single axon to isolate axon injury events, which is not possible *in vivo* in the mammalian adult CNS. WT adult retina explants display very little neurite outgrowth, which corresponds exactly to the failure of regeneration in the mature WT CNS. Our system can be used to compare axon regrowth following single axon injury in WT versus PTEN^–/–^ axons to study various cellular and molecular events downstream activation of mTOR. Alternatively, WT adult retina explants could be used as a baseline system to test different drugs that potentiate or block cellular pathways pharmacologically. This could be done before or after laser guided ablation as a therapeutic approach for CNS axon regeneration.

### Growth Cone Dynamics and Guidance in Adult Axons

While axons from the PNS are able to form a growth cone within hours after lesion (Pan et al., 2003), CNS axons fail to achieve this step and instead, the tip of the lesioned axon is sealed by a retraction bulb (Ertürk et al., 2007). It is believed that this abortive attempt to form a functional growth cone is part of the mechanism underlying the failure of the mature CNS to regenerate and is the limiting step to reach axon growth (Verma et al., 2005; Bradke et al., 2012; Edwards and Hammarlund, 2014). Therefore, it is crucial to identify molecular and cellular regulators of growth cone formation in CNS axons. In this context, adult retina explant cultures are highly handful to address this question. To study growth cone reformation after axonal lesion, we combined live imaging and laser ablation of a single axon. In a previous study we showed that the structural protein DCLK2 (doublecortin like kinase 2) enhances axon regeneration by inducing growth cone formation through actin cytoskeleton stabilization (Nawabi et al., 2015). This set-up allows to analyze regenerating growth cones in live and in detail, and thus to study how mature axon growth cones respond to their environment. Furthermore, the behavior of growing axons toward their target is still elusive (Pernet and Schwab, 2014). While modulation of neuronal intrinsic abilities to promote axon regrowth has led to long distance regeneration and stimulation of neuronal activity has resulted in some functional recovery (Bei et al., 2016; Lim et al., 2016), the formation of functional circuits still remains challenging. Indeed, long distance growth is always associated with strong guidance defects. Most axons are blocked at the chiasm, some are turning back or others misproject into the brain (Pernet and Schwab, 2014; Crair and Mason, 2016; He and Jin, 2016). Those observations are true with any system of long distance regeneration (Sun et al., 2011; Luo et al., 2013; Pernet et al., 2013a, b; Belin et al., 2015). Therefore, deciphering the interaction between regenerating axons, especially their growth cones, and molecules expressed in the environment will be key to drive these axons to functional targets. Large-scale analysis of purified RGC that are regenerating reveal the expression of several cues involved in axon guidance (Sun et al., 2011; Norsworthy et al., 2017). Guidance cues are also expressed in functional targets of RGC, for example with the recent identification of guidance molecules in the suprachiasmatic nucleus based on single-cell transcriptomics (Wen et al., 2020). In this context, adult explants induced for axon regeneration are a valuable *ex vivo* tool to assess regenerating RGC response to guidance cues by setting up paradigms used for developing axons (Gundersen and Barrett, 1979; Raper and Kapfhammer, 1990; Falk et al., 2005).

### Axonal Transport Features in Response to Injury

Moreover, even if molecular pathways that control axon regeneration have been uncovered, the underlying mechanisms are still difficult to elucidate. For example, discrepancy in axonal transport has been linked to neurodegenerative diseases in many studies but remains to be fully characterized (Millecamps and Julien, 2013; Kiryu-Seo and Kiyama, 2019). Accelerating mitochondria transport leads to axon regeneration by supplying healthy mitochondria and rescuing energy deficits in the injured axon (Zhou et al., 2016). Overexpression of enhancers of mitochondria movement, such as the protein Armcx1, triggers axon regeneration in the optic nerve (Cartoni et al., 2016). In addition, mitochondria depolarize at the lesion site, as can be observed with tetramethylrhodamine ethyl ester (TMRE) loss of staining (Zhou et al., 2016). Timing of analysis in our *ex vivo* system can be adapted to look closer at this phenomenon, for example by recording fluorescence recovery hours rather than minutes after laser guided ablation.

In our *ex vivo* system, mitochondria could be labeled using viral vectors that express MitoDsRed or using a specific tracking dye added extemporary into the culture media. Here we showed that in mature intact axons mitochondria are stationary, as observed *in vivo* for the majority of mitochondria (Misgeld et al., 2007; Smit-Rigter et al., 2016; Misgeld and Schwarz, 2017), although intravital imaging of mitochondrial axonal transport in mouse RCG shows some active transport near the soma in intact conditions (Takihara et al., 2015) that we did not studied *ex vivo* as we analyzed distal regions of the axon. In contrast to adult axons, mitochondria are motile in developing neurons (Faits et al., 2016; Lewis et al., 2016). This opposite behavior in developing and mature axons could create a bias when it comes to study the contribution of mitochondria during regeneration, again showing the importance of an *ex vivo* model as close to *in vivo* conditions as possible. In adult axons, we observed that mitochondria are stationary in intact conditions and following the lesion. It would be interesting to test pathways known to regulate their motility and recruitment and address the outcome upon lesion (Han et al., 2016; Zhou et al., 2016; Cartoni et al., 2017; Lee et al., 2019).

Interestingly, other organelles such as lysosomes show a high dynamics in mature axons, with a tendency for retrograde movement that correlates with their maturation (Gowrishankar et al., 2017). Upon lesion we observed a significant decrease of their velocity. Yet, it is now understood that correct lysosome polarized movement in the axon is essential for maintaining global axon functioning, including growth cone dynamics (Farías et al., 2017). More generally, lysosome transport deficits are associated with many neurodegenerative disorders (Zheng et al., 2010; Ferguson, 2018; Lie and Nixon, 2019). Thus, the almost complete interruption of lysosome dynamics caused by axonal injury may strongly impair the regenerative capacity of adult CNS axons. It has been previously shown that axon lesion induces the disruption of actin and microtubule cytoskeleton (Bradke et al., 2012). Most of the organelles use the microtubules as highways to navigate within the cells (Guedes-Dias and Holzbaur, 2019). Therefore, it is not surprising that both lysosomes and mitochondria are stationary upon lesion as microtubules depolymerize. It would be interesting to address organelle movement when the cytoskeleton is preserved upon taxol treatment, which stabilizes microtubules, or upon overexpression of microtubule-associated and actin regulating proteins such as DCLK2.

## Conclusion

In this study we show that adult retina explant cultures are the ideal *ex vivo* system to explore the molecular and cellular events that occur upon axon lesion in the mature CNS. This model recapitulates the *in vivo* phenotype and offers to characterize finely adult CNS axons at a single axon level, in a biological set-up far more relevant and accurate to the field of CNS repair than embryonic neuronal cultures. We described a non-exhaustive series of experimental applications of this model, such as characterization of growth cone behavior and study of organelle transport in the axon. It is critical to understand these events to find new targets to achieve repair of the mature nervous system and formation of functional circuits. Importantly, dissecting these mechanisms is crucial to design translational approaches and propose a treatment for patients suffering from CNS traumatic injuries, but also from neurodegenerative disorders, which raise similar questions of neuroprotection and neuroregeneration in the adult CNS.

## Data Availability Statement

The original datasets generated for this study are available upon request to the corresponding authors.

## Ethics Statement

All animal care and procedures have been approved by the Ethics Committee of the Grenoble Institut Neurosciences (project number 201612161701775) and by the French Ministry of Research (project number APAFIS#9145-201612161701775v3) in accordance with French and European guidelines.

## Author Contributions

HN and SB: conceptualization, methodology, supervision, and funding acquisition. JS and CD: performed the experiments. HN, SB, JS, and CD: original draft and editing. FA: mouse colony handling and technical assistance. All authors contributed to the article and approved the submitted version.

## Conflict of Interest

The authors declare that the research was conducted in the absence of any commercial or financial relationships that could be construed as a potential conflict of interest.

## Supplementary Material

Supplementary Figure 1 Quantification of axon regeneration in optic nerves whole-mount. **(A–C)** Comparison of optic nerves from control (PTEN^*fl/fl*^ + AAV2-Plap), PTEN-deleted (PTEN^*fl/fl*^ + AAV2-Cre) and PTEN/SOCS3 co-deleted c-myc-overexpressing (PTEN^*fl/fl*^/SOCS3^*fl/fl*^ + AAV2-Cre + AAV2-CNTF + AAV2-c-myc) conditions, at different distances from the injury site. Data are expressed as means ± s.e.m. Unpaired *t*-tests. ^∗^*p* < 0.05, ^∗∗^*p* < 0.01, ^∗∗∗^*p* < 0.001, ^****^*p* < 0.0001. **(D)** Principle of axon regeneration analysis in whole transparent optic nerves. Representative confocal picture of CTB-labeled optic nerve whole-mount from a PTEN^*fl/fl*^ mouse with prior intravitreal injection of AAV2-Cre. Red stars indicate the injury site. The confocal picture is annotated with ticks spaced 100 μm along the optic nerve. CTB-555 intensity is measured at defined steps (colored lines) of optic nerve, with background measurement in a region with no axon regeneration (gray line). At each defined step, the area under the curve is measured and normalized to optic nerve width. Values are normalized to the maximal intensity value of all defined steps. Finally, the background value is subtracted. Scale bar: 500 μm.

Supplementary Figure 2 Quantification of axon growth in adult retina explant cultures. **(A–C)** Comparison of explant cultures from control (PTEN^*fl/fl*^ + AAV2-Plap), PTEN-deleted (PTEN^*fl/fl*^ + AAV2-Cre) and PTEN/SOCS3 co-deleted + c-myc-overexpressing (PTEN^*fl/fl*^/SOCS3^*fl/fl*^ + AAV2-Cre + AAV2-CNTF + AAV2-c-myc) conditions, at different distances from the explant. Data are expressed as means ± s.e.m. Unpaired *t*-tests. ^∗^*p* < 0.05, ^∗∗^*p* < 0.01, ^∗∗∗^*p* < 0.001, ^****^*p* < 0.0001. **(D)** Representative pictures of adult retina explants after 2 weeks in culture, from control (PTEN^*fl/fl*^ + AAV2-Plap), PTEN-deleted (PTEN^*fl/fl*^ + AAV2-Cre) and PTEN/SOCS3 co-deleted + c-myc-overexpressing (PTEN^*fl/fl*^/SOCS3^*fl/fl*^ + AAV2-Cre + AAV2-CNTF + AAV2-c-myc) conditions. Axons are labeled with anti-β Tubulin III (TUJ1) antibody. Scale bar: 500 μm. **(E)** Example of Sholl analysis for quantification of number of axon intersect.

Supplementary Video 1 Growth cone of axon in adult retina explant culture (DIC illumination).

Supplementary Video 2 Standard lasercut of single axon (DIC illumination).

Supplementary Video 3 Strong lasercut of single axon (DIC illumination).

Supplementary Video 4 Actin dynamics in growth cone (Lifeact-tdTomato).

Supplementary Video 5 Mitochondria in intact axon (MitoDsRed).

Supplementary Video 6A Mitochondria in intact axon (MitoTracker).

Supplementary Video 6B Mitochondria in axon post-cut (MitoTracker).

Supplementary Video 7A Lysosomes in intact axon (LysoTracker).

Supplementary Video 7B Lysosomes in axon post-cut (LysoTracker).

## Abbreviations

AAV2: Adeno-associated virus type 2
BSA: bovine serum albumin
CNS: central nervous system
CNTF: ciliary neurotrophic factor
Cre: Cre recombinase
CTB-555: Alexa 555-conjugated cholera toxin B
DCLK2: doublecortin like kinase 2
GFP: green fluorescent protein
JAK/STAT: Janus Kinase/Signal Transducer and Activator of Transcription
KLF: krüppel like factors
mTOR: mammalian target of rapamycin
PBS: phosphate buffered saline
PFA: paraformaldehyde
Plap: placental alkaline phosphatase
PNS: peripheral nervous system
PTEN: Phosphatase and TENsin homolog
PTEN^*fl/fl*^: PTEN-floxed
Q: linear flow rate
RBPMS: RNA-binding protein with multiple splicing
RGC: retinal ganglion cells
SOCS3: Suppressor of cytokine signaling 3
SOCS3^*fl/fl*^: SOCS3-floxed
TMRE: tetramethylrhodamine ethyl ester
TRITC: Tetramethylrhodamine
Vma: anterograde velocity
Vmr: retrograde velocity
WT: wild-type
YFP: yellow fluorescent protein.
