# Supplementary figures and images for: Adult Mouse Retina Explants: From ex vivo to in vivo Model of Central Nervous System Injuries
Source: Front Mol Neurosci. 2020 Nov 25;13:599948. doi: 10.3389/fnmol.2020.599948 (PMC7723849; doi:10.3389/fnmol.2020.599948)

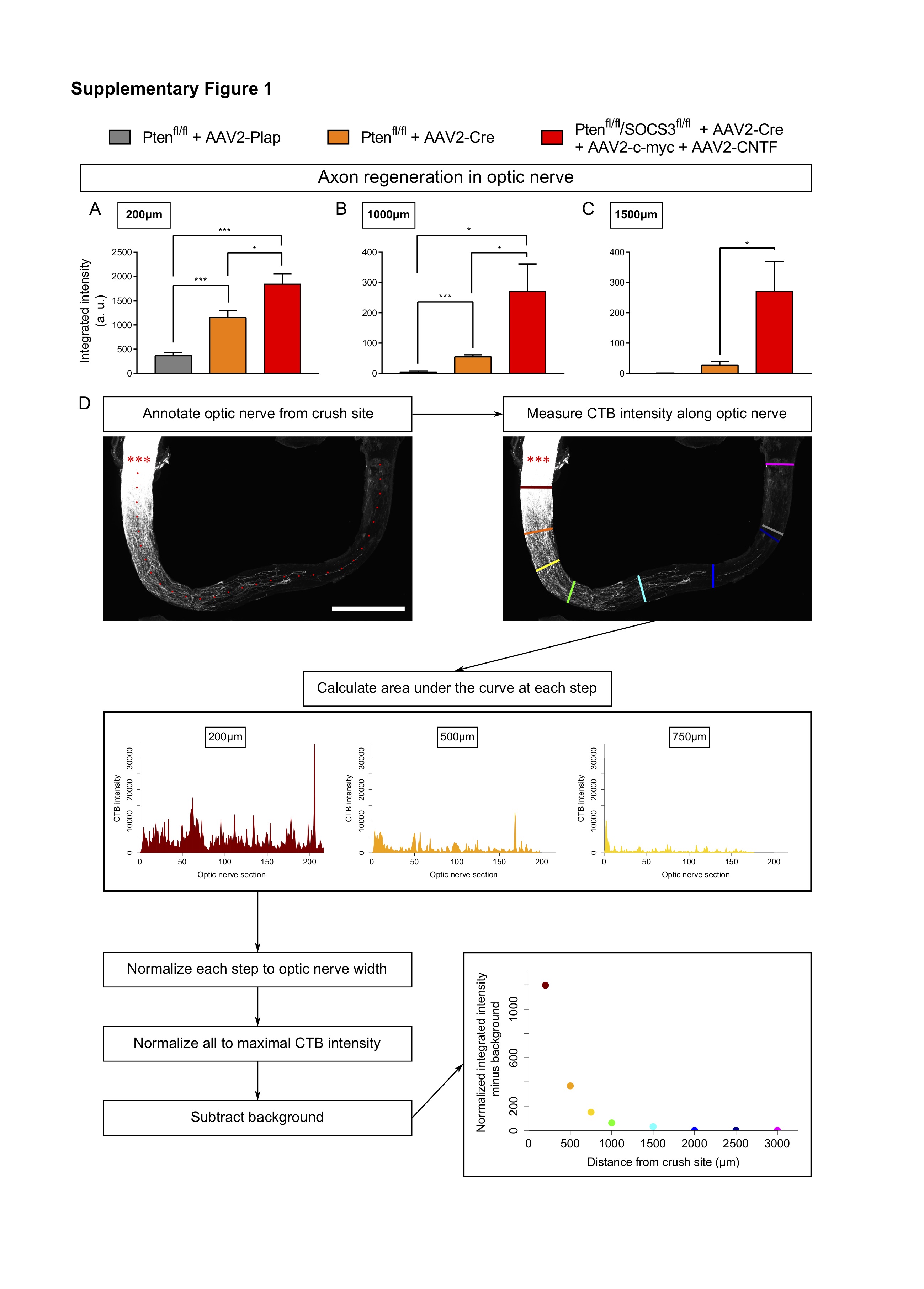

Supplement: Supplementary Figure 1 — Quantification of axon regeneration in optic nerves whole-mount. (A–C) Comparison of optic nerves from control (PTENfl/fl + AAV2-Plap), PTEN-deleted (PTENfl/fl + AAV2-Cre) and PTEN/SOCS3 co-deleted c-myc-overexpressing (PTENfl/fl/SOCS3fl/fl + AAV2-Cre + AAV2-CNTF + AAV2-c-myc) conditions, at different distances from the injury site. Data are expressed as means ± s.e.m. Unpaired t-tests. ∗p < 0.05, ∗∗p < 0.01, ∗∗∗p < 0.001, ****p < 0.0001. (D) Principle of axon regeneration analysis in whole transparent optic nerves. Representative confocal picture of CTB-labeled optic nerve whole-mount from a PTENfl/fl mouse with prior intravitreal injection of AAV2-Cre. Red stars indicate the injury site. The confocal picture is annotated with ticks spaced 100 μm along the optic nerve. CTB-555 intensity is measured at defined steps (colored lines) of optic nerve, with background measurement in a region with no axon regeneration (gray line). At each defined step, the area under the curve is measured and normalized to optic nerve width. Values are normalized to the maximal intensity value of all defined steps. Finally, the background value is subtracted. Scale bar: 500 μm. [file Image_1.JPEG]

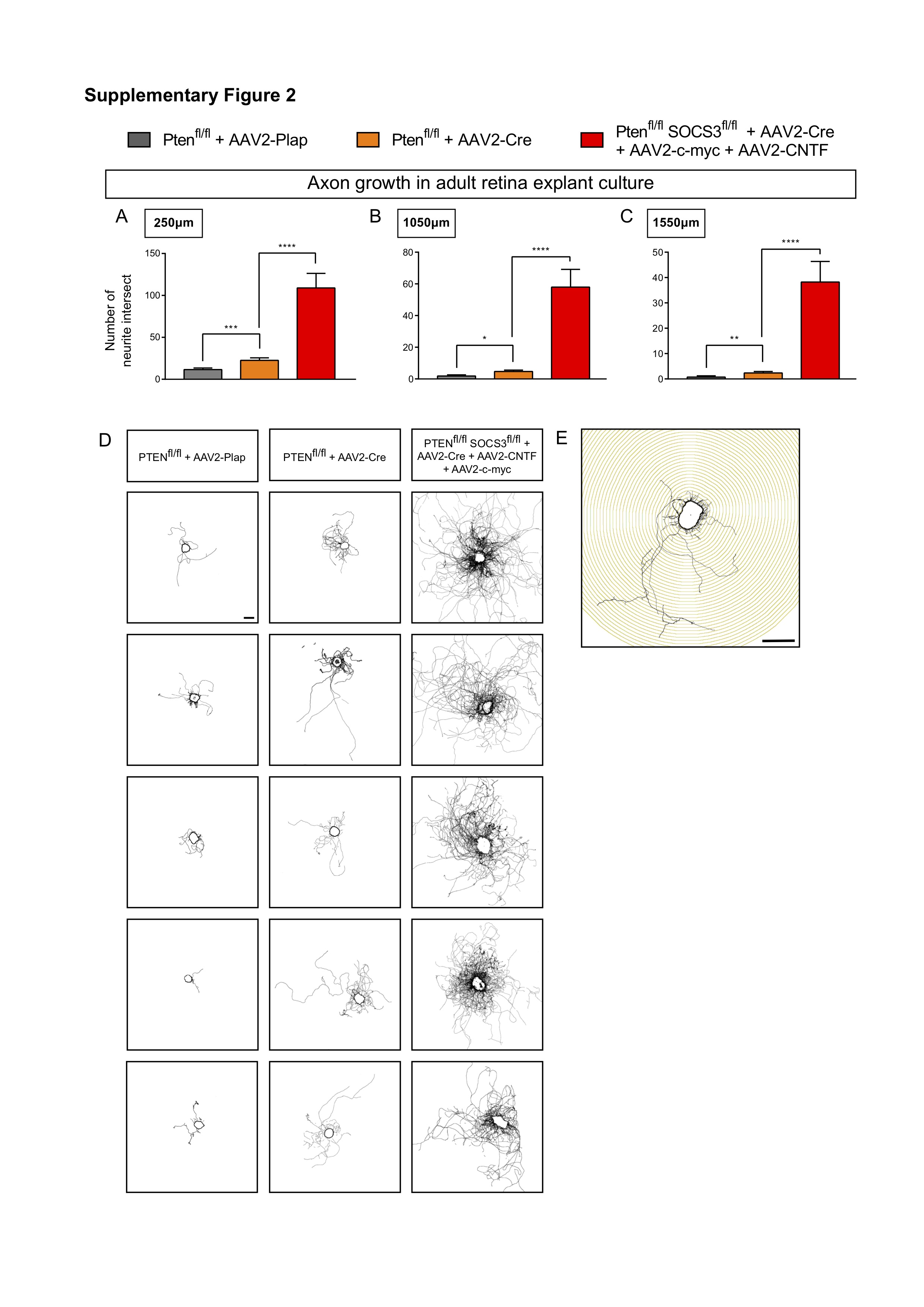

Supplement: Supplementary Figure 2 — Quantification of axon growth in adult retina explant cultures. (A–C) Comparison of explant cultures from control (PTENfl/fl + AAV2-Plap), PTEN-deleted (PTENfl/fl + AAV2-Cre) and PTEN/SOCS3 co-deleted + c-myc-overexpressing (PTENfl/fl/SOCS3fl/fl + AAV2-Cre + AAV2-CNTF + AAV2-c-myc) conditions, at different distances from the explant. Data are expressed as means ± s.e.m. Unpaired t-tests. ∗p < 0.05, ∗∗p < 0.01, ∗∗∗p < 0.001, ****p < 0.0001. (D) Representative pictures of adult retina explants after 2 weeks in culture, from control (PTENfl/fl + AAV2-Plap), PTEN-deleted (PTENfl/fl + AAV2-Cre) and PTEN/SOCS3 co-deleted + c-myc-overexpressing (PTENfl/fl/SOCS3fl/fl + AAV2-Cre + AAV2-CNTF + AAV2-c-myc) conditions. Axons are labeled with anti-β Tubulin III (TUJ1) antibody. Scale bar: 500 μm. (E) Example of Sholl analysis for quantification of number of axon intersect. [file Image_2.JPEG]
